# Recent Advances in Natural Functional Biopolymers and Their Applications of Electronic Skins and Flexible Strain Sensors

**DOI:** 10.3390/polym13050813

**Published:** 2021-03-06

**Authors:** Ziying Wang, Zongtao Ma, Jingyao Sun, Yuhua Yan, Miaomiao Bu, Yanming Huo, Yun-Fei Li, Ning Hu

**Affiliations:** 1 Tianjin Key Laboratory of Electronic Materials and Devices, School of Electronics and Information Engineering, Hebei University of Technology, 5340 Xiping Road, Tianjin 300401, China; wangzy@hebut.edu.cn (Z.W.); 202021901036@stu.hebut.edu.cn (Z.M.); 202031903012@stu.hebut.edu.cn (J.S.); 201931903011@stu.hebut.edu.cn (Y.Y.); 202031903086@stu.hebut.edu.cn (M.B.); 201931903012@stu.hebut.edu.cn (Y.H.); 2 Center for Advanced Laser Technology, Hebei University of Technology, Tianjin 300401, China; 3 Hebei Key Laboratory of Advanced Laser Technology and Equipment, Tianjin 300401, China; 4 State Key Laboratory of Reliability and Intelligence Electrical Equipment, Hebei University of Technology, Tianjin 300130, China; 5 National Engineering Research Center for Technological Innovation Method and Tool, School of Mechanical Engineering, Hebei University of Technology, Tianjin 300401, China

**Keywords:** natural biopolymers, electronic skins (e-skins), flexible strain sensors, functionality, biocompatible

## Abstract

In order to replace nonrenewable resources and decrease electronic waste disposal, there is a rapidly rising demand for the utilization of reproducible and degradable biopolymers in flexible electronics. Natural biopolymers have many remarkable characteristics, including light weight, excellent mechanical properties, biocompatibility, non-toxicity, low cost, etc. Thanks to these superior merits, natural functional biopolymers can be designed and optimized for the development of high-performance flexible electronic devices. Herein, we provide an insightful overview of the unique structures, properties and applications of biopolymers for electronic skins (e-skins) and flexible strain sensors. The relationships between properties and sensing performances of biopolymers-based sensors are also investigated. The functional design strategies and fabrication technologies for biopolymers-based flexible sensors are proposed. Furthermore, the research progresses of biopolymers-based sensors with various functions are described in detail. Finally, we provide some useful viewpoints and future prospects of developing biopolymers-based flexible sensors.

## 1. Introduction

Recently, flexible and wearable electronics devices have shown tremendous application potential in the fields of Internet of Things (IoT), sensor, energy, biomedical systems, artificial intelligence (AI) and smart robots [1,2,3]. Compared with traditional electronic equipment, flexible electronics have many advantages such as portability, flexibility, extensibility, low manufacturing cost, unique deformability and so on. As key parts of flexible electronics, electronic skins (e-skins) and flexible strain sensors can be widely used for humans’ daily activities monitoring, human-machine interfaces, AI, and the prevention and rehabilitation of human diseases [4,5]. The flexible sensors include optical-, electronical-based sensors and some sensors based on other technologies. Optical fiber sensors are widely used in flexible and wearable devices due to their advantages such as multiplexing capabilities, compact size and electromagnetic immunity [6,7]. These advantages provide an intrinsically safe solution for underwater, intrusive and harsh environment applications [8,9]. Flexible and wearable electronical-based sensors exhibit excellent features, such as portability, high sensitivity, a simple preparation process and low cost, which benefit from the electromechanical properties of their conductive materials. So far, multiple flexible conductive materials, for instance, metal nanoparticles and metal oxides [10,11], one dimension (1D) carbon nanotubes (CNTs) [12,13], two dimensional (2D) transition metal dichalcogenides [14,15], graphene [16,17] and carbide nanosheets [18,19], three dimensional (3D) metal-organic frameworks (MOFs) [20,21] and conductive polymers [22,23] have been used for the fabrication of flexible sensors. Despite the fact these materials have been widely investigated, their intrinsic low mechanical properties, poor stability, non-biocompatibility, non-biodegradability and toxicity have limited their applications.

**Figure 1:**
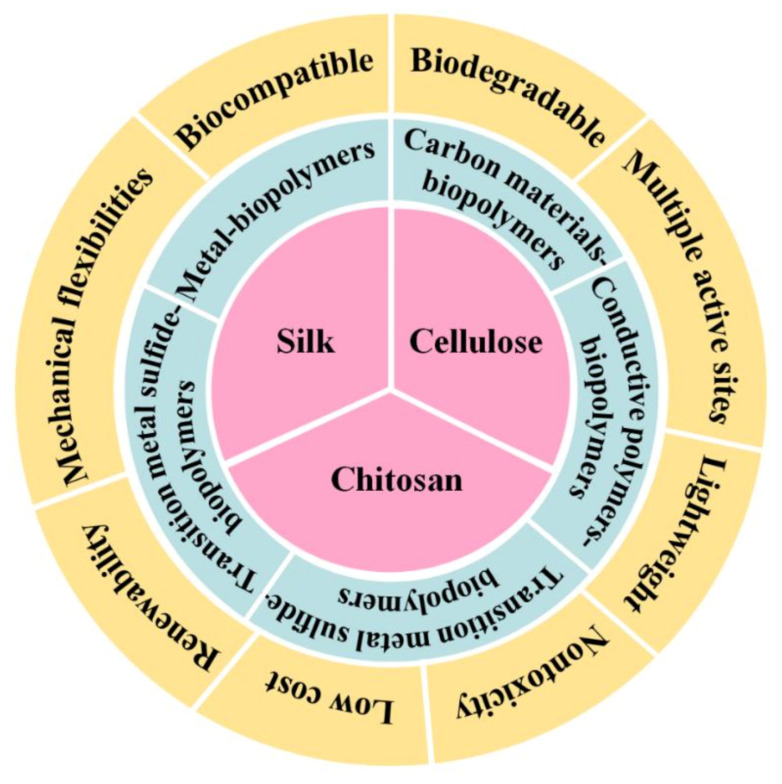
The advantages of three natural biopolymers and their composites for flexible sensors.

Natural biopolymers are naturally derived from bio-based materials, e.g., a kind of raw materials (such as microorganisms, plants, fats, sugars, proteins and their components) by biological and chemical synthesis methods [24]. In comparison with synthetic nanomaterials, biopolymers possess many unique advantages, such as natural abundance, robust structures, hydrophilicity, water solubility, multiple active sites, self-cleaning, lightweight, mechanical flexibilities, biocompatibility, biodegradability, non-toxicity, renewability, low cost, etc.) (Figure 1). Biopolymers are promising materials for developing environmentally friendly and sustainable flexible sensors [24,25]. At first, the special layered topographic characteristics of biopolymers provide adjustable elastic modulus, offering customizable mechanical properties to conform bending interface and dynamic surface [26]. Specifically, some unique properties of biopolymers, including self-cleaning and self-healing properties, allow biopolymer-based sensors to avoid interference from external influences (e.g., humidity, temperature, dust, etc.) and improve the test accuracy. In addition, the low cost, light weight, non-toxicity, and biological characteristics can accommodate green and large-scale fabrication of flexible sensors, and reduce the discomfort of long term attachment on the human skin surface. This kind of sensors have shown significant potentials in human health monitoring. At last, the existence of abundant functional groups in biopolymers, such as hydroxyl, carboxylic, amino groups, etc. [25] is conducive to further functionally modify biopolymers and endow biopolymers more functionalities.

In this paper, we summarize the recent development of natural biopolymers with various nanostructures as functional and sensing materials in flexible electronics. The functional design strategies and fabrication technologies for biopolymers-based flexible sensors are proposed by analyzing the structures and properties of biopolymers. Then, the research progresses of biopolymers-based sensors with various functions in flexible electronics devices are summarized. In the end, the current challenges and trends of biopolymers-based sensors are described and discussed.

## 2. Structures and Properties of Biopolymers

Recently, natural biopolymers have attracted extensive research interest in flexible electric device applications [27]. Natural biopolymers also show outstanding advantages in flexible sensors due to their superior performance, similar to that of biopolymers. These excellent characteristics result from their special structures. Therefore, it is necessary to have an in-depth understanding of the structure, the physicochemical and biological properties of biopolymers, and even the relationship between structures and properties. This might optimally utilize biopolymers in the design of flexible sensors and further expand the potential requests of biopolymers-based flexible electronics. Herein, we come up with three of the most common biopolymers, silk, cellulose, and chitosan, respectively, and then concisely introduce the structures and properties of biopolymers.

### 2.1. Silk

Natural silk is as a continuous long silk fibroin fiber formed by the solidification of the silk liquid secreted by the mature silkworm. Silk is famous for its prominent mechanical flexibility, biocompatibility, nontoxicity, etc., which is commonly used as e-textiles and suture materials to fabricate sensors [28]. The silks are composed of fibroin fibers wrapped in sericin. Sericin is usually removed via the process of degumming to get elastic and biocompatible silk fibroin (SF). SF contains several amino groups, including serine, alanine, and glycine, which are connected by peptide bond. Then, the polypeptide chains are formed, which consist of a heavy (H) type chain (H-chain) and a light (L) type chain (L-chain) [29]. By the interactions of intermolecular and intramolecular, the hydrophobic repetitive regions of H-chain can formulate β-crystallites with nanoscale, which are responsible for the fascinating robust mechanical performance [30]. The mechanical properties of SF can benefit from its unique hierarchical structure, which derive from the ordered and interlocking arrangement of SF [2]. In addition, the water soluble, good biocompatible and biodegradable features of SF are attributed to fibrous protein, which contains amino acids. These special performances make silk a good candidate for the development of wearable electronics and biomedical [31,32]. Furthermore, through chemical modification and combined effect with conductive materials, silk-based materials, such as silk-based hydrogels, fibers, nanowires and films were used to develop flexible electronic devices, which would be discussed in detail in the third section.

### 2.2. Cellulose

Cellulose is one of typical polysaccharide, which is made up of ordered *β*-(1,4)-linked D-glucose units, and has plenty of hydroxyl (–OH) groups, resulting in inter- and intramolecular interaction bonding. Then, strong and tremendous hydrogen-bond networks are constructed among polymeric chains, which can endow the high crystallinity and high axial stiffness of cellulose microfibrils [33]. In plants, the cellulose chains from macromolecular are assembled into basic fibrils with lengths of over several hundred nanometers. The microfibrils are made up of rectangular arrays of these elementary fibrils, which stack up together to form plant cell walls [34]. The complicated and integral structures between lignin and cellulose carbohydrates give the microfibrils with supernormal structure stability and 1D nanostructure features. It is notable that cellulose can be processed into various nanocellulose, including cellulose-based nanofibrils, nanocrystals, and bacterial cellulose (BC), that are used for various applications [35]. In comparison with cellulose nanocrystals, cellulose nanofibrils and BC exhibit long length and large specific surface area, resulting in perfect mechanical robustness [33].

### 2.3. Chitosan

Chitosan is a linear biopolymer and also a derivative of chitin produced by deacetylation. It possess unique chemical and physical properties by controlling the length of chitin chain and the degree of chemical processing, such as the depolymerization and the deacetylation [36]. The structure of chitosan contains some amino groups and hydroxyl groups, that are readily available for crosslinking [37]. The complexation between cations and anions is easily happened [38], resulting in the formation of chitosan with various morphologies, including nanoparticles, films, and hydrogels have been developed. Due to their excellent biocompatibility, biodegradability and controllable mechanical properties, abovementioned chitosan have been widely studied in the fields of flexible electronics [39,40,41].

## 3. Applications of Biopolymers-Based E-Skins and Flexible Strain Sensors

Based on the above excellent characteristics of biopolymers, current progresss in the application of biopolymers in wearable electronics and flexible sensors are concluded. Here is the point that the fabrication of natural flexible material systems and then generalize relevant applications in flexible sensors and wearable electronics.

### 3.1. Biopolymer-Based E-Skins

Soft, flexible and stretchable e-skins are an array of sensors (including pressure sensors, temperature sensors, humidity sensors, strain sensors, etc.) that can mimic the properties of biological skin and sense the external environment. Recently, many kinds of functional materials such as conducting polymers [42,43,44], carbon-based materials [45,46] and low-dimensional materials [47,48] were selected to serve the high-performance wearable e-skins sensors owing to the excellent mechanical performances (e.g., flexibility, low modulus and stretchability). However, the practical applications of most reported e-skins based on above nanomaterials is limited due to the unknown bio-toxicity and biocompatibility.

**Figure 2:**
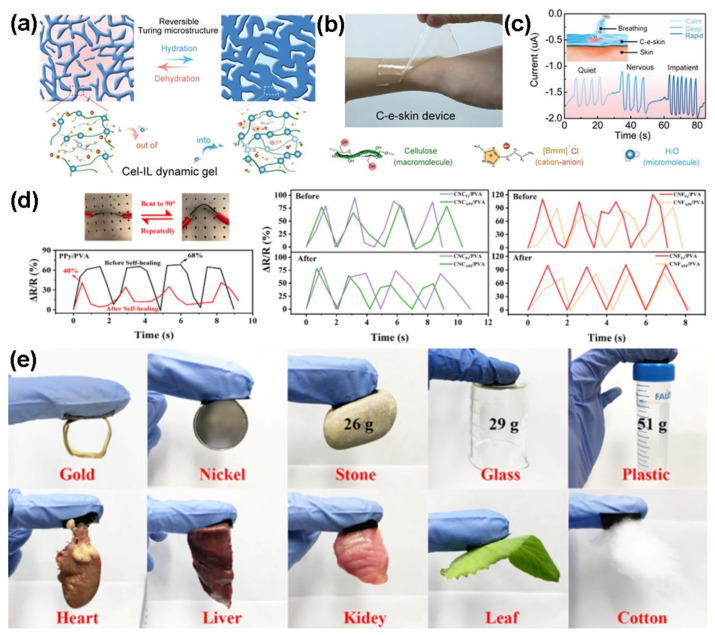
(**a**) Tunable and reversible turing-pattern microstructures of Cel-IL dynamic gel, (**b**) a piece of stretchable C-e-skin can attach and conform to a human external wrist, (**c**) current waveforms of C-e-skin can sense breathing (calm, deep, and rapid breathing, respectively) [49] Reproduced with permission from ref. [49]. Copyright 2020, Elsevier; (**d**) change in strain sensitivity of PPy/PVA (left), CNC_Fe_/PVA (middle purple), CNC_APS_/PVA (middle green), CNF_Fe_/PVA (right red), and CNF_APS_/PVA (right orange) before and after self-healing, (**e**) self-adhesive test of CNF_Fe_/PVA on different substrates [50]. Reproduced with permission from ref. [50]. Copyright 2019, American Chemical Society.

Natural biopolymers have characterized structures and fascinating properties and are promising materials for e-skins. As shown in Figure 2a, Zhao et al. presented cellulose-based e-skins containing by using an ion gel, including cellulose, ions and water (Cel-IL dynamic gel). The e-skins exhibited the microstructure like bramble with ionic conductivity, favorable adhesion and fast self-healing properties (Figure 2a) [49]. Further, the biomimetic e-skins were extremely sensitive and recognizable to some mechanical strains and human movements (Figure 2b,c), making them promising for monitoring human movement status. Han et al. have prepared self-healable and conductive polypyrrole-coated cellulose based nanocomposites (cellulose nanocrystals and nanofibers) for biocompatible e-skin sensor systems, which were functionalized by FeCl_3_ and polyvinyl alcohol (PVA) [50]. The combination of iron coordination bonds and H-bonds could improve the mechanical robustness, electrical conductivity and self-healing capability of cellulose nanocomposites. The nanocomposites possessed promising sensing properties for the monitoring of various human motions, such as finger bending, swallowing and pulse. Even though the nanocomposite films were damaged, they could still show positive mechanical strain (72.0–76.3%) and conductive recovery after half an hour (Figure 2d). Additionally, the PVA-coated cellulose composites also exhibited excellent adhesion on various substrates (Figure 2e).

**Figure 3:**
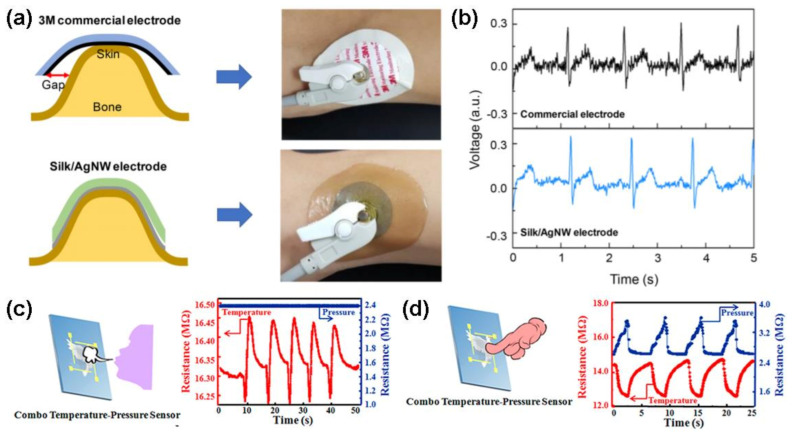
(**a**) Schematics and photos of conformal attachment of AgNW/silk electrode, (**b**) ECG signals captured by electrodes attached to bump of the wrist. The nonconforming nature of the commercial electrode induces a large amount of noise in the signal. Reproduced with permission from [51]. Copyright 2018, American Chemical Society. Schematic illustration and simultaneous sensing performance of the combo e-skin sensor under various stimuli (**c**) exhaling stimulus and (**d**) finger pressing stimulus. Reproduced with permission from [52]. Copyright 2017, American Chemical Society.

Jo et al. have reported on highly stretchable and conformal e-skins, which combine silk protein hydrogel and metallic nanowire networks. The e-skins provide good extensibility and stability under the condition of hydration [51]. These properties allow the e-skins to detect electrophysiological, strain sensing and electrochemical signals, simultaneously (Figure 3). Wang et al. have fabricated dual functional e-skins for the monitoring temperature and pressure sensing information, which was assembled by silk-nanofiber-derived carbon fiber membranes (Silk CFM) [52]. The temperature sensor showed enhanced sensitivity of 0.81%/°C. The strain sensor presented high sensitivity (gauge factor: ~8350 at 50% strain), which can sense the stimulation of subtle pressure. Wang et al. have constructed highly sensitive e-skin arrays based on multiwalled CNTs-sunflower pollen (MWCNTs-SFP) hybrids [53]. According to their research results, the e-skins sensor exhibited low detection limit (1.6 Pa) and good stability (>25,000 cycles) due to strong hierarchical structure and the tunable elastic modulus of hollow spheres.

### 3.2. Biopolymer-Based Flexible Strain Sensors

Flexible strain sensors have attracted growing attentions owing to their valuable applications in monitoring of human physiological signals, the interaction between human and machine, as well as wearable sensors [54,55]. Compared with synthetic polymers, biopolymers are biocompatible and innoxious, which contribute to the improvement of the recycling performance for flexible sensors.

#### 3.2.1. Silk-Based Flexible Strain Sensors

**Figure 4:**
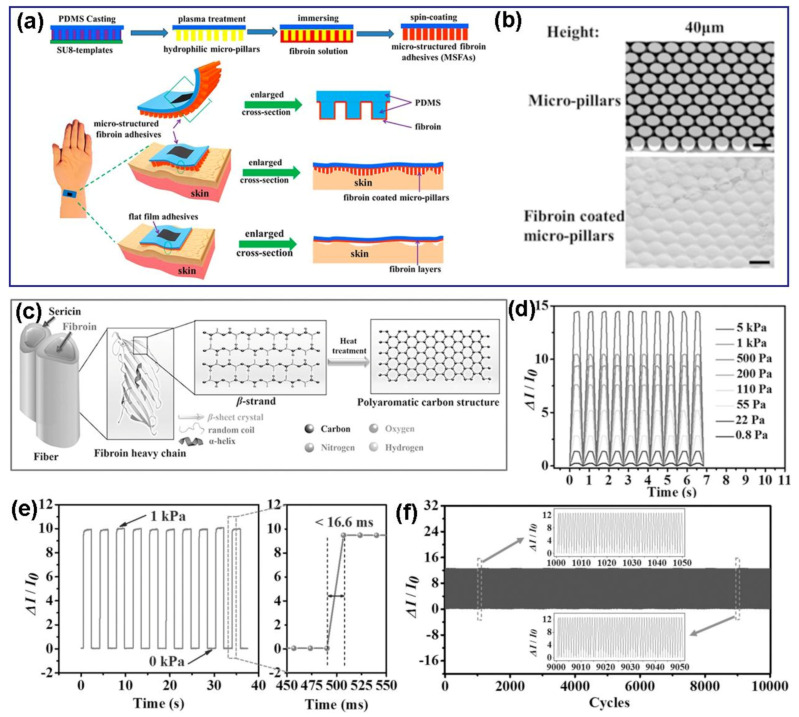
(**a**) Fabrication process of microstructured fibroin adhesive (MSFA) and the schematic of an MSFA on the skin of the wrist, (**b**) scanning electron microscopy (SEM) images of bare (top) and fibroin (bottom) coated micropillars with a diameter of 80 μm and heights of 40 μm. Reproduced with permission from [58]. Copyright 2020, American Chemical Society; (**c**) the schematic illustration structure of pristine silk and carbonized silk, (**d**) multiple cycles of pressure response under 10 Pa–5 kPa, (**e**) the pressure response at high frequency and the response time within 16.6 ms, (**f**) the durability test of the pressure sensor over 10,000 loading-unloading cycles at a frequency of 0.5 Hz under an applied pressure of 2.5 kPa. Reproduced with permission from [59]. Copyright 2017, WILEY-VCH.

Silk is one of representative natural biopolymers, which has gained tremendous attention in the application of flexible stretchable sensors [56,57]. Liu et al. have prepared a silk fibroin-based adhesive with micropillar structures on polydimethylsiloxane (PDMS) substrate (Figure 4a,b), which exhibited dependable and stabilized bonding force on the surfaces of skin in the condition of different humidity. The microstructure of fibroin adhesive has an increasing potential for applications in various epidermal electronic sensors [58]. Wang et al. have developed a highly sensitive strain sensor based on carbonized silk nanofibers (CSNs) (Figure 4c), which exhibited superior performance in detecting human physiological signals. The improved sensing performance is attributed to the nitrogen doping carbon nanofiber network structure of CSNs [59]. Figure 4d–f demonstrate that the obtained strain sensor presented short response time (<16.7 ms), supersensitivity (34.47 kPa^−1^), high durability (>10,000 cycles) and low detection limit (0.8 Pa).

**Figure 5:**
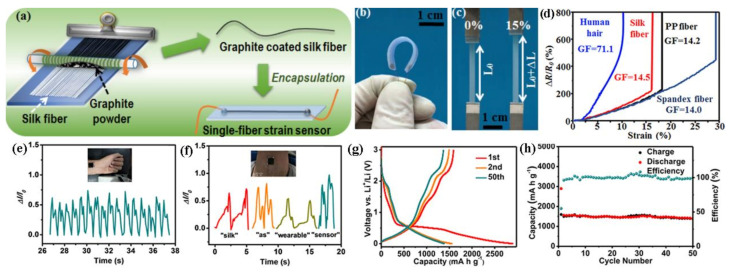
(**a**) Fabrication of sheath-core structured graphite/silk strain sensors through a dry-Meyer-rod-coating process, (**b**) photograph of the flexible strain sensor, (**c**) photograph of the strain sensor subjected to strain of 0% and 15%, (**d**) comparison of the sensitivity and elongation of sheath-core graphite/fiber strain sensors with different core fibers, including hair, silk, polypropylene (PP), and Spandex fibers. Reproduced with permission from [60]. Copyright 2016, American Chemical Society; (**e**) the response of the MoS_2_/CSFs sensor for monitoring healthy adults’ wrist pulse, (**f**) the response curves for the sound signal when the volunteer spoke “silk” “as” “wearable” “sensor”, (**g**,**h**) electrochemical properties of MoS_2_/CSFs as the anode in Li-ion batteries. Reproduced with permission from [61]. Copyright 2020, American Chemical Society.

In terms of improving conductivity and flexibility of pure SF, many researchers have tried to combine the good conductive materials with SF. For examples, Zhang et al. have used a one-step dry-Meyer-rod-coating process to construct sheath-core-structured fiber strain sensors, where graphite flakes were as the sheath and silk fibers were as the core (Figure 5a–c) [60]. As shown in Figure 5d, the gauge factor of this strain sensor is 14.5 and working strain range is 15%. Meanwhile, the core-shell structured strain sensors fabricated by other materials include human hair, polypropylene (PP) fiber, and spandex fiber exhibited different gauge factor values, respectively. The graphite/silk fiber-based strain sensor can realize the measurement of multidirectional strain in the field of wearable strain sensors. Besides, inspired by the unique structure of the faceplate of sunflowers, Zhang’s group has constructed a wearable sensor based on the layered structure via in-situ grown MoS_2_ nanosheets on carbonized silk fabrics (MoS_2_/CSFs), that can be used not only to monitor feeble physiology signals (Figure 5e,f) but also to serve for lithium-ion batteries (Figure 5g,h) [61]. Liu et al. have prepared conducting graphene oxide (GO) and silk fabrics, followed by chemical reduction [62]. The resistivity and conductivity of silk fabrics using regenerated silk fibroin as a glue can be obviously improved, which can satisfy the needs of flexible electronic textiles. It is a big challenge to prepare biocompatible and high-performance flexible sensing films due to the large-scale production and chemical toxicity. Wang et al. have designed flexible and biocompatible silk fibroin and MXene biocomposites with 3D network structure, which possessed low elastic modulus (1.22 MPa), short response time (40 ms), low detection limit (9.8 Pa) and perfect stability (3500 cycles), severally [63].

**Figure 6:**
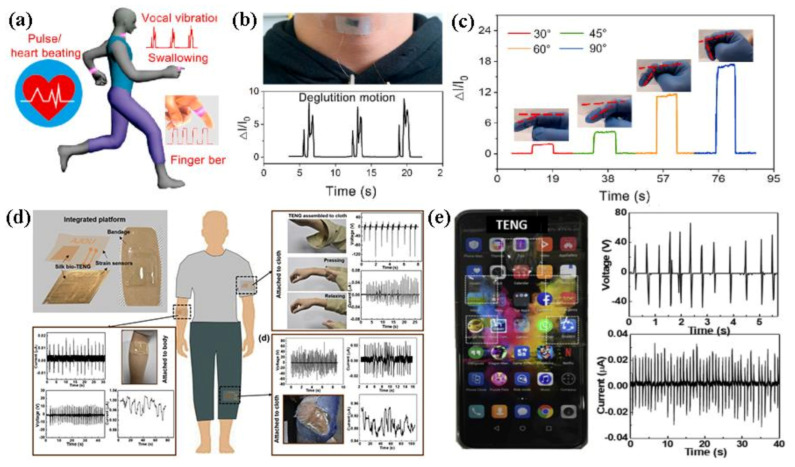
(**a**) Overview of sensing applications of the SF/Mxene flexible sensor for the detection of various physiological human motions, (**b**) Photograph of the SF/Mxene flexible sensor attached onto the throat and response signals of deglutition motion, (**c**) the response of SF/Mxene flexible sensor in monitoring finger bending under different angles. Reproduced with permission from [63]. Copyright 2020, Elsevier; (**d**) schematic diagram and photograph images of the integrated strain sensor and the bio-TENG on a bandage, and the applications of the integrated devices to measure various human motions, (**e**) photograph images and outputs of the bio-TENG devices attached to a cell phone. Reproduced with permission from [64]. Copyright 2019, Elsevier.

The SF/MXene films provided applicable safe and high-performance flexible sensors in the application of detecting various physiological human motions (Figure 6a–c). Gogurla and his co-workers have reported a self-actuated device, which contained a strain sensor and a bio-triboelectric nanogenerator (TENG), which were prepared by using silver nanowires (AgNWs) embedded in silk film to detect human movement information and collect energy from them (Figure 6d,e) [64]. As a strain sensor, it can show high sensitivity with a gauge factor about 30 and detect the flexural motion of the knuckles. The finger-contact can power the silk-based TENG and produce high power density with 2 mW/cm^2^, which can actuate light-emitting diodes (LEDs). The silk-based TENG possessed optical transparency, which made it possible to use as a touch sensor in the field of flexible electronics.

#### 3.2.2. Cellulose-Based Flexible Strain Sensors

**Figure 7:**
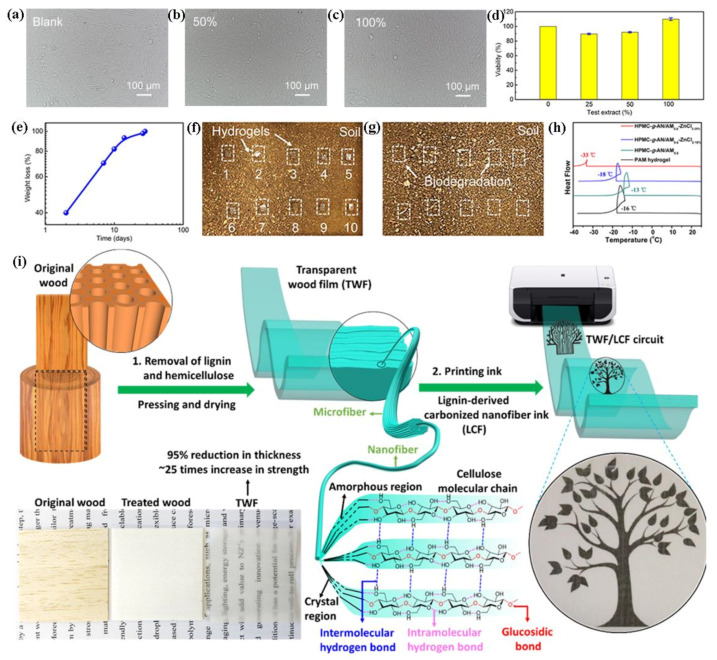
(**a**–**c**) Optical micrographs of NIH3T3 cells exposed to blank, 50% and 100% of hydrogel extract, (**d**) Viability of NIH3T3 cells after incubating with hydrogel extracted DMEM at different concentration, (**e**) weight loss of okara hydrogels during degradation time, pieces of okara cellulose hydrogels in the soil on the first day (**f**) and day 28 (**g**) in the biodegradable test. Reproduced with permission from [69]. Copyright 2019, Nature Publishing Group; (**h**) The DSC curves of the PAM, HPMC-*g*-AN/AM_0.6_, HPMC-*g*-AN/AM_0.6_-ZnCl_2–15%_, and HPMC-*g*-AN/AM_0.6_-ZnCl_2–25%_ hydrogel at the temperature range from 25 °C to 40 °C. Reproduced with permission from [70]. Copyright 2020, American Chemical Society; (**i**) illustration processing of TWF for flexible electronics application [73]. Reproduced with permission from ref. [73]. Copyright 2020, American Chemical Society.

Cellulose is one kind of worldwide abundant natural biopolymer, which can be extracted from some of plants (including trees, bamboos, agricultural crops, etc.) and microorganisms (including *Acetobacter xylinum*, *Komagataeibacter*, *Enterobacter*, *Rhodococcus*, *Sarcina,* etc.) [65,66]. Cellulose are suitable for the manufacture of flexible electronics because of their chemical uniqueness, easy processability, renewability, and biodegradability. Significant cellulose-based materials have been synthesized by reasonably controlling the surface/interface chemistry and adopting advanced processing technologies, which bring new opportunities for the new-generation flexible sensors [67,68]. Cui et al. extracted cellulose from okara (soybean waste)followed by the fabrication of non-cytotoxic cellulose hydrogels with excellent mechanical properties (Figure 7a–c) and biodegradability (Figure 7d–f) [69]. In addition, the conductive cellulose-based hydrogel (HPMC-*g*-AN/AM_x_-ZnCl_2-y_) was synthesized by grafting acrylamide and acrylonitrile copolymers onto the cellulose chains [70]. The cellulose hydrogel exhibited good electric conductivity of 1.54 S/m, remarkable tensile strength of 160 kPa, stretchability of 1730%, and superior antifreezing performance even under the temperature of −33 °C (Figure 7h), which exhibited high-performance in monitoring human activities.

Wood is a green, environmentally and biodegradable natural material, which is used to extract cellulose or lignin. Moreover, by tailoring the hierarchical nanostructure of wood, flexible sensors can be fabricated [71,72]. As shown in Figure 7i, Fu et al. designed a transparent wood film (TWF)-based flexible electronics circuit, which was printed with the conductive ink composed of lignin-derived carbon nanofibers [73]. The flexible wood-based film was strong with a Young’s modulus of 49.9 GPa and a tensile strength of 469.9 MPa in the fiber direction. Chen et al. have reported a facile strategy to prepare an elastic wood-derived carbon aerogel from CNFs and lignin [74]. The carbon aerogel exhibited high compressibility under a strain up to 95% and high sensitivity from 0 to 16.89 kPa. Moreover, the wood-derived carbon aerogel was incorporated into a flexible symmetric supercapacitor. Besides, Song et al. have prepared a biocompatible, biodegradable, flexible 3D porous membrane directly extracting from the natural wood, which displays the application in 3D bio-scaffold for cell growth [75].

**Figure 8:**
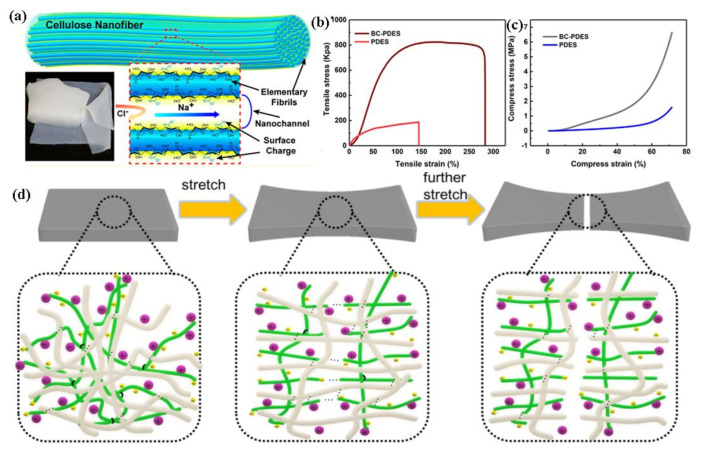
(**a**) The TEMPO treatment process significantly imparts negative surface charge on the cellulose nanofibers for highly selective cation transport and photographs of the hydrated pristine BC. Reproduced with permission from [76]. Copyright 2018, American Chemical Society; (**b**) typical tensile stress/strain curves, (**c**) compressive stress/strain curves of BCs-PDESs and PDESs, (**d**) possible energy dissipation mechanism of the double polymer chain network under stretching. Reproduced with permission from [77]. Copyright 2020, American Chemical Society.

In contrast to wood-based cellulose, bacterial cellulose (BC) demonstrates excellent properties including high crystallinity, and water-holding capacity. Wang et al. have reported a bio-compatible ion conductor, which is made of 2,2,6,6-tetramethylpiperidine-1-oxyl radical (TEMPO)-treated BC [76]. The TEMPO-treated BC film showed a 3D interconnected network, containing large negatively charged surface groups (Figure 8a), which can be constructed an ultrasensitive flexible sensor for wearable health monitor applications. Yin et al. have developed an innovative BC-based ionic conductor. The polymerizable deep eutectic solvents were addressed to solve the dehydration or deliquescence issues due to the sensitivity toward humidity inevitably [77]. The BC-PDESs ionic conductor exhibited tensile strength of 8 × 10^5^ Pa (Figure 8b) and compressive strength of 6.68 × 10^6^ Pa (Figure 8c) because of the 3D nanofiber structure and strong interfacial interaction (Figure 8d). In addition to these, some 2D materials and conductive polymers were combined with cellulose to develop high-performance flexible sensors [78,79,80]. For instance, BC/multiwall carbon nanotubes (MWCNTs) [78], BC/MXene (Ti_3_C_2_) nanosheets [79], BC/polyvinyl alcohol/CNTs/carbon black [80] were assembled as flexible strain sensors to monitor human body bio-signals.

Cellulose can be incorporated with functional inorganic and organic materials to develop high-performance flexible sensors [81,82,83,84,85,86,87]. For example, a conductive Ag nanowires (AgNWs)/cellulose nanofibers (CNFs) nanopaper was applied in flexible sensors by a solution blending and vacuum filtration method [81]. A tensile strain sensor with the AgNWs/CNFs nanopaper sandwiched between two thermoplastic polyurethane (TPU) films presented an ultralow detection limit of 0.2%, good stability and durability after 500 strain cycles. A bending strain sensor was also prepared by adhering AgNWs/CNFs nanopaper onto a TPU film, exhibiting stable linearly sensing behavior under tension and compression strain.

**Figure 9:**
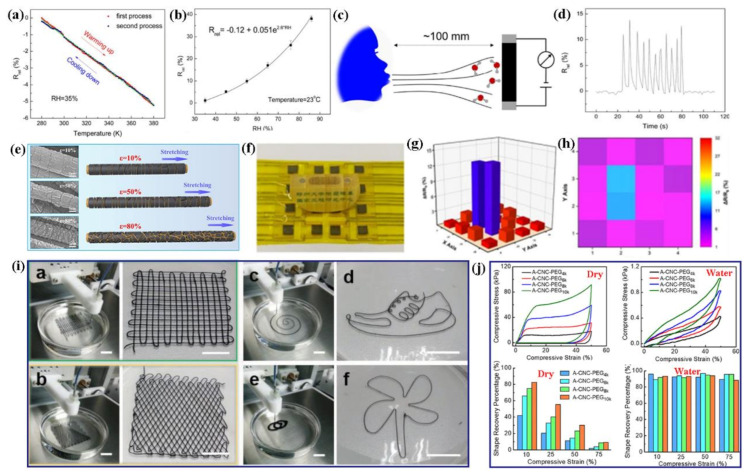
(**a**,**b**) Sensitivity of rGO/cellulose composite to temperature and humidity, (**c**) schematic for in-situ monitoring electrical resistance changes of rGO/cellulose composite film placed at a distance up to 100 mm from one’s nose, (**d**) dependence of R_rel_ on time for in-situ monitoring human breathing cycles of inhalation and exhalation. Reproduced with permission from [83]. Copyright 2018, Royal Society of Chemistry; (**e**) In-situ SEM images and the corresponding schematic illustration for the microstructure change of the strain sensor during the tension process, (**f**–**h**) photographs of different items onto the e-skin and the corresponding spatial pressure distribution mapping based on the resistance variation. Reproduced with permission from [84]. Copyright 2020, Royal Society of Chemistry; (**i**) optical images of the 3D-printed TOCNFs/Ti_3_C_2_ fabrics with woodpile, fishing net structures and other designed geometric structures. Reproduced with permission from [85]. Copyright 2019, WILEY-VCH; (**j**) compressive stress-strain curves of aerogels for CNCs-PEG aerogels in the dry state and in water, shape recovery percentage of aerogels of CNCs-PEG aerogels under different compressive strains: in the dry state and in water. Reproduced with permission from [86]. Copyright 2020, American Chemical Society.

Cellulose/carbon materials have also been explored for constructing flexible strain sensors [82,83]. Xie et al. have prepared non-cytotoxic cellulose/CNT composite films, which possessed a high tensile strain of 17.7%. They are perspective candidates for tactile information collection [82]. Cellulose/graphene nanocomposite films were constructed by dissolving cellulose and graphene oxide (GO) mixture in aqueous solution; while GO was reduced to RGO by chemical reduction technique [83]. Surprisingly, the rGO/cellulose nanocomposite can be utilized in multifunctional sensor responding to humidity, temperature, and stress/strain changes (Figure 9a–d).

In addition to carbon materials, 2D transition metal carbides Ti_3_C_2_T_x_ MXene is considered to be promising functional material applied in sensors. Li et al. have fabricated wearable strain sensors based on MXene/cellulose nanocrystals coated TPU nonwoven fabrics (MC@p-NWF) [84]. The MC@p-NWF-based strain sensor displayed ultrahigh sensitivity with gauge factor of 3405, a wide sensing range of 83%, and a low detection limit of 0.1%. Furthermore, the high sensitivity of the MC@p-NWF-based sensor was mainly attributed to the increased contact area of the NWF interfaces under compression strain. As expected, the MC@p-NWF based sensor can be applied for the applications (Figure 9e–h), such as human physical signals collection and motion monitoring. Cao et al. have fabricated the flexible textiles and fibers by employing a 3D printing technique with the inks of Ti_3_C_2_ MXene and 2,2,6,6-tetramethylpiperidine-1-oxyl radical (TEMPO)-mediated oxidized cellulose nanofibrils (TOCNFs) (Figure 9i) [85]. Compared to traditional synthetic fibers, the Ti_3_C_2_/TOCNFs fibers and textiles exhibited strong response to photonic, electrical, and mechanical stimulus. In order to improve the elasticity of the cellulose nanocrystals (CNCs)-based aerogels, the CNCs was cross-linked with elastic poly ethylene glycol (PEG) [86]. The CNCs/PEG aerogel with chemical-bond cross-linking and a H-bond network possessed a dry-state modulus of 0.80 MPa in and a we-state modulus of 0.87 kPa (Figure 9j).

#### 3.2.3. Chitosan-Based Flexible Strain Sensors

**Figure 10:**
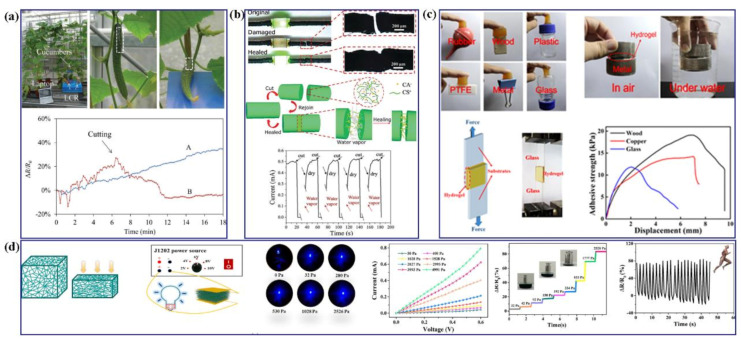
(**a**) Fruit growths of cucumbers were being measured. LCR meters were used to measure the real-time resistance values of the sensors written on a cucumber. Reproduced with permission from [87]. Copyright 2017, WILEY-VCH; (**b**) optical microscopy images of a CS/CNT fiber at original, damaged and healed states to turn on or off a LED light bulb, schematic illustration of the healing process of a CS/CNT fiber exposed to water vapor, repeated healing and recovery of electrical properties for five cuts on the fiber. Reproduced with permission from [90]. Copyright 2018, Royal Society of Chemistry; (**c**) self-adhesion properties of CS/PAA/TA@CNC-60, hydrogels tightly adhered between skin and various substrate surfaces, including rubber, wood, plastic, PFTE, metal and glass, schematic illustration of lap shear test, representative curves of adhesion shear force versus displacement for hydrogels with various substrates (wood, copper and glass). Reproduced with permission from [94]. Copyright 2019, American Chemical Society; (**d**) stress-sensing models of PANI/BC/CH aerogel, the schematic diagram of the LED light was applied to test the electrical response of the PANI/BC/CH aerogel under a power supply of 2 V in a series circuit, response of the LED light varies with the applied pressure on the PANI/BC/CH aerogel, current-voltage (I-V) curves of PANI/BC/CH aerogel under different pressures, the curve of ΔR/R_0_ versus time when PANI/BC/CH aerogel-based sensor was attached on the bottom of a shoes as a pedometer for walking. Reproduced with permission from [96]. Copyright 2019, WILEY-VCH.

Chitosan (CS) is an increasingly used polysaccharide with superior biocompatibility, biodegradability, nontoxicity and excellent mechanical properties, which make it a promising natural sensing material for flexible strain sensors. Recently, a CS-based water ink was used to fabricate stretchable stain sensors through writing the ink followed by drying for about 15 min [87]. Notably, the sensor could be stretched up to 60% and showed a gauge factor of 64 under the strain of 1–8%. Besides, CS-based sensors could be written on cucumber fruits; while the cucumber growths were being monitored in real time by measure the resistance changes of the sensors (Figure 10a). These results showed that the fabrication of “green” sensor reduced the energy consumption for wearable monitoring. Except as mentioned above, other materials such as CNTs, carbon black (CB), conductive polymers, and graphene are used in combined with chitosan to fabricate the flexible strain sensors [88,89,90,91,92]. For examples, CS/CB nanocomposite sponges were fabricated via combining solution-mixing and freeze-drying methods, which presented sensitive strain sensing performance [88]. Carbon materials with good conductivity, high elasticity and extensibility are suitable candidates for flexible sensors. Luo et al. have proposed a simple and effective method to build a compressible carbon aerogel from multiwalled CNTs by employing CB as a binder [89]. CB can link multiwalled CNTs into continuous lamellas and result in stable 3D structures. The multiwalled CNTs/CB hybrids aerogel could suffer from a high compression strain of 90% with only 8% deformation at 50% strain even after 50,000 strain-release cycles. Wu et al. have reported novel multifunctional nanocomposites based on CS and MWCNTs microstructured fibers with electrical conductivity and self-healing ability [90]. Specifically, the self-healing time of the composite is within seconds (Figure 10b). More importantly, the CNT/CS microstructured fibers were exposed to water vapor and then their mechanical characteristics were restored after healing the broken sacrificial bonds. Xia et al. have fabricated a functional material composed of CS, graphene oxide and polyacrylic acid (PA), which can withstand a harsh environment [91]. The gel-based strain sensor demonstrates rapid response of 40 ms, superior stretchability of more than 1000%, and good stability, effectively increasing the durability in applications of wearable devices and soft robot systems.

In recent years, conductive polymer hydrogels have drawn much attention in flexible devices due to their self-adhesion, excellent electronic and mechanical properties [92,93]. Cui et al. have reported the stretchable, conformable, antibacterial and self-adhesive CS/PA network hydrogels, which were prepared by in-situ polymerization methods with tannic acid coated cellulose nanocrystal (TA@CNC) acting as nanofillers [94]. The hydrogels showed good controllable mechanical properties, which were easily adjusted with fracture stress and fracture strain range (370–800%). What is more, the hydrogels can be attached onto wooden, plastic, glass and human skin substrates, etc. because of their high adhesion capability (Figure 10c). Wei et al. have prepared a chitosan/silicotungstic acid-poly(acrylamide) (CS/SiW-PAM) crosslinked hydrogel, which possessed some excellent properties, such as antibacterial, adhesive, self-healing effects and so on [95]. The abovementioned hydrogel also displayed promising repeatable adhesion on various hard and soft substrates, highly conductivity, favorable self-healing as well as antibacterial performances. Finally, Huang et al. have fabricated a piezoresistive sensor based on the flexible multifunctional aerogels via adding polyaniline (PANI) into bacterial cellulose/chitosan (BC/CS) hybrids [96]. The PANI/BC/CH based sensor displayed excellent electromechanical performance (Figure 10d). LED light was used to test the electrical response of PANI/BC/CH hybrids under different levels of pressure. The luminance of the LED light was adjusted with the mechanical deformations of the hybrids. Furthermore, the sensor based on PANI/BC/CH, as a pedometer, was placed on the sole of shoes, which showed the stable and repeatable response of resistance in the running process. Indeed, CS/conductive polymer hydrogels provide effective strategies for development of functional soft materials in the applications of flexible wearable devices.

## 4. Conclusions and Perspectives

In this review, several natural biopolymers and their applications in e-skins and flexible strain sensors are proposed and summarized to meet rising requirement of green electronics, soft integration and biocompatible manufacturing technologies. Biopolymers have many merits, such as unique structures, mechanical flexibility, biocompatibility, biodegradability, nontoxicity, renewability, etc. In order to obtain desirable and high-performance sensing properties, various advanced technologies and methods for preparing functional biopolymers and biopolymers composites have been developed.

Despite the great advances has been achieved in recent research of biopolymers and biopolymer-based sensors, there are some challenges in realizing the practical application of biopolymers in flexible electronics. For instance, firstly, although natural biopolymers have been developed as non-toxic, biocompatible and biodegradable platforms for flexible electronics, other incorporated components (e.g., metal nanoparticles/nanowires, carbon materials, conductive polymers) have limitations in this regard. Secondly, further mechanistic research and synthetic methods for novel biopolymers need to be developed so as to endow biopolymers with new special and outstanding properties, for example, electronic conductivity, bioactivity, thermostability, 3D conformal properties and high ion mobility on skins and curved surface; Finally, reasonable surface/interface engineering techniques, fabrication technology of substrates and multifunctional integration of biopolymers-based flexible sensors need to be investigated and enhanced. Undoubtedly, biopolymers provide an exciting strategy to exploit new functional flexible electronics that are of great significance for improving their applications in green and sustainable and smart electronic devices.

## Data Availability

The data presented in this study are available on request from the corresponding author.
